# Identification of mosquitoes (Diptera: Culicidae): an external quality assessment of medical entomology laboratories in the MediLabSecure Network

**DOI:** 10.1186/s13071-018-3127-7

**Published:** 2018-10-23

**Authors:** Frédéric Jourdain, Marie Picard, Tatiana Sulesco, Nabil Haddad, Zoubir Harrat, Samer Saleh Sawalha, Filiz Günay, Khalil Kanani, Taher Shaibi, Denys Akhramenko, M’hammed Sarih, Enkelejda Velo, Lusine Paronyan, Igor Pajovic, Chafika Faraj, Irakli Sikharulidze, David Putkaradze, Jelena Maric, Golubinka Bosevska, Elizabeta Janceska, Ali Bouattour, Afrim Hamidi, Kurtesh Sherifi, Bülent Alten, Dušan Petrić, Vincent Robert

**Affiliations:** 1 Research Unit MiVEGEC, French National Research Institute for Sustainable Development, IRD-CNRS-Montpellier University, Montpellier, France; 2 Institute of Zoology, Ministry of Education, Culture and Research, Chisinau, Moldova; 3 Laboratory of Immunology and Vector Borne Diseases, Lebanese University, Fanar, Lebanon; 4 Laboratoire Éco-épidémiologie Parasitaire et Génétique des Populations, Institut Pasteur d’Algérie, Algiers, Algeria; 5 Ministry of Health, Public Health General Directorate, Environmental Health Department, Vector Control Unit, Ramallah, Palestine; 6 Faculty of Science, Biology Department, Ecology Section, Vector Ecology Research Group Laboratories, Hacettepe University, Ankara, Turkey; 7 Parasitic and Zoonotic Diseases Department, Vector-Borne Diseases Programmes Manager, MOH, Amman, Jordan; 8 Reference Laboratory of Parasites & Vector Borne Diseases, NCDC Libya, and Zoology Department, Faculty of Science, University of Tripoli, Tripoli, Libya; 9 State Body I.I. Mechnikov Ukrainian Anti-Plague Research Institute of Ministry of Health, Odessa, Ukraine; 10 Laboratoire des Maladies Vectorielles, Institut Pasteur du Maroc, Casablanca, Morocco; 11 Control of Infectious Diseases Department, Institute of Public Health, Tirana, Albania; 12 Vector-borne and Parasitic Diseases Epidemiology Department, NCDC, Ministry of Health, Yerevan, Armenia; 13 Biotechnical Faculty, University of Montenegro, Podgorica, Montenegro; 14 Laboratoire d’Entomologie Médicale, Institut National d’Hygiéne, Rabat, Morocco; 15 Zooentomology Laboratory, National Center for Disease Control and Public Heath, Tbilisi, Georgia; 16 P.I. Veterinary Institute of the Republic of Srpska “Dr. Vaso Butozan”, Banja Luka, Bosnia and Herzegovina; 17 Laboratory for virology and molecular diagnostics, Institute of Public Health of R. Macedonia, Skopje, Former Yugoslav Republic of Macedonia; 18 Université de Tunis El Manar, Institut Pasteur de Tunis, LR11IPT03 Service d’Entomologie Médicale, Tunis, Tunisia; 19 Faculty of Agriculture and Veterinary Sciences, University of Prishtina, Pristina, Republic of Kosovo; 20 Faculty of Science, Biology Department, Ecology Section, Vector Ecology Research Group Laboratories, Hacettepe University, Ankara, Turkey; 21 Faculty of Agriculture, Laboratory for Medical and Veterinary Entomology, University of Novi Sad, Novi Sad, Serbia; 22 French National Research Institute for Sustainable Development, Research Unit MiVEGEC, IRD-CNRS-Montpellier University, Montpellier, France

**Keywords:** EQA medical entomology, Mediterranean Sea, Mosquito species, Capacity building, Quality control, International network

## Abstract

**Background:**

Identification of vectors is of prime importance in the field of medical entomology for both operational and research purposes. An external quality assessment of mosquito identification capacities was carried out within the MediLabSecure Network, which is composed of laboratories located in 19 countries close to the European Union around the Mediterranean and Black seas.

**Methods:**

A set of blind samples consisting of 7 or 8 adult mosquitoes and 4 larvae was given to each participant laboratory. In all, 138 adult mosquitoes and 76 larvae of different species were distributed for genus and species identification.

**Results:**

All identifications were exclusively morphology based. Overall, 81% of identifications were correct at the genus level, 64% at the species level. The results were highly varied among the 19 participating laboratories. The levels of correct identifications were: 100% (three laboratories), 90–95% (four laboratories), 50–75% (six laboratories) and < 50% (six laboratories).

**Conclusions:**

This evaluation showed the need to maintain efforts in capacity building and quality control in the field of medical entomology and, more specifically, in the morphological identification of the Culicidae.

**Electronic supplementary material:**

The online version of this article (10.1186/s13071-018-3127-7) contains supplementary material, which is available to authorized users.

## Background

**Fig. 1:**
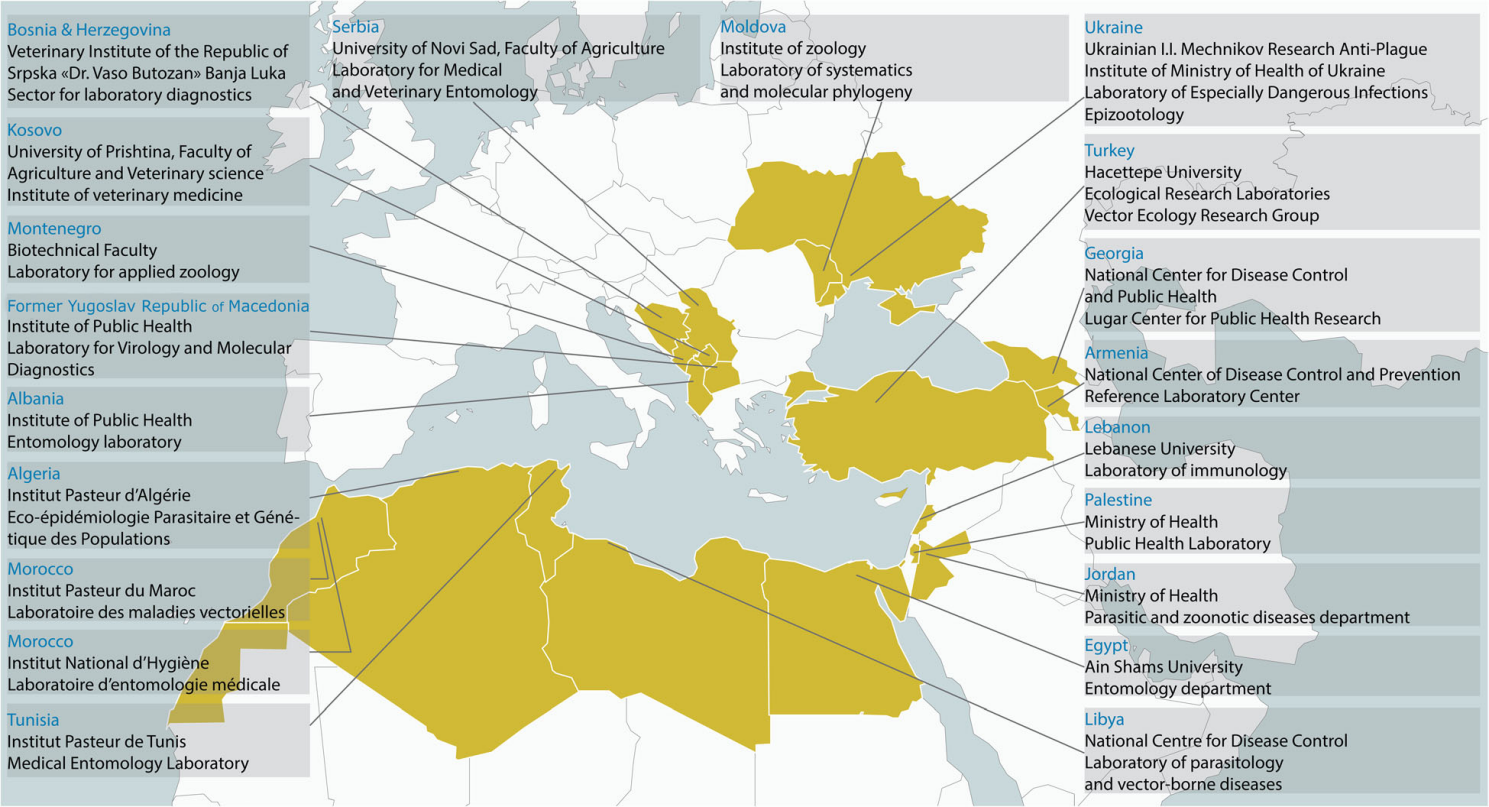
Map of the member countries of MediLabSecure and the locations of the laboratories constituting the medical entomology network

The MediLabSecure project aims to develop capacities of preparedness and response to viral emergencies by establishing an integrated network covering the fields of human virology, animal virology, medical entomology and public health in 19 non-European Union countries in the Mediterranean and Black Sea regions (Fig. 1). One medical entomology laboratory in each country (two in one of the countries) were selected for inclusion in the network.

In the broad sense, vectors encompass any organism involved in the transmission of an infectious agent. More specifically, in medical and veterinary entomology, vectors designate any haematophagous arthropod which actively transmits an infectious pathogen from an infected host to a new host [1]. In a given zoological group, vector species are ordinarily outnumbered by other non-vector species. For instance, among the 540 species of mosquito in the subfamily Anophelinae, about 60 species are recognised vectors of *Plasmodium*, the agent of malaria [2]. Identification of arthropod species, both vectors and non-vectors, is a core capacity in the field of medical and veterinary entomology. Accurate identification of a species involved in transmitting a pathogen is essential for a proper understanding of the mechanisms that govern any biological system. Knowing which arthropod species transmit vector-borne diseases is also vital to providing species-specific focused control programmes and to correctly identifying disease risk and exposure [3].

Determining the quality of taxonomic data is a matter of recent concern. Where data of unknown quality are used, they naturally lead to conclusions of unknown quality. It is, therefore, crucial that uncertainty associated with taxonomic identification is documented and reported. This issue is central to any monitoring programme in biology [4–6] as well as in ecology [7].

Different approaches to mosquito identification are available: morphological, molecular (PCR and nucleic acids sequencing), proteomics tools, isozyme analysis, etc. [8, 9]. However, for historical and technical reasons, morphological identification is still the reference method for both research purposes and operational surveillance as it requires little technical equipment, is easy to implement in the field and is inexpensive even when large numbers of individuals need to be identified. The method has three principal limitations, the first being that it relies on expert entomologists performing the identification, especially when many species are present in the collection area. The second limitation resides in the level of preservation of the morphological characters used in the sample handling process. The third concerns species complexes and a few morphologically similar species where identification should be based on more than one developmental stage, which is not always accessible.

Most traditional morphological identification keys are organised in a series of alternative statements, which may be dichotomous (every choice has two alternatives) or polytomous (two or more options at each choice). In practice, most of them are a mixture of dichotomous and polytomous sequential choices. Importantly, they are structured as single-access keys because they trace a unique path in the decision tree. An alternative to this type of fixed sequence of choice is the multi-access key (or matrix key), where the sequence choices are largely up to the user [10]. Dichotomous keys are printable and multi-access keys are the most suitable tools for computer-aided identification. Dichotomous keys tend to be used linearly; identification choices must be made stepwise and missing out a step or choice can result in a sample being misidentified. Multi-access keys have the advantage of allowing steps to be missed out, so it may still be possible to identify a damaged sample or to look for other morphological characters when one cannot be clearly determined. Within these two types of key, the dichotomous keys by Becker et al. [11] and the computer-aided MosKeyTool [12] are among the most commonly used for identifying mosquitoes in the Euro-Mediterranean Sea region.

External quality assessments (EQA) have for decades been regularly implemented in medical laboratories to appraise a wide range of analyses [13]. They are essential for quantifying the quality of routine laboratory work and ensuring the quality of biological test results, which are often the basis for clinical decision making. A quick search in PubMed using the phrase “External Quality Assessment” OR “External Quality Assurance” OR “External Quality Control” found 2883 occurrences (accessed 15 May 2018). However, we found no published EQAs on insects as nuisances or vectors, with the exception of Chaki et al. [14], who dealt with operational field quality control issues rather than vector determination. A self-assessment of the quality of mosquito identification was therefore performed within the medical entomology activities of MediLabSecure. While EQAs are often carried out in quality control in medical laboratories, we present here what is, to the best of our knowledge, the first EQA in the field of identifying arthropods of medical interest.

## Methods

The EQA was designed and coordinated by Medical Entomology Work Package leaders of the MediLabSecure Network, none of whom belonged to any of laboratories that took part in the EQA. A full list of the 19 participating laboratories is given in Additional file 1: Table S1.

A set of blind samples consisting of seven or eight adult specimens plus four stage IV larvae in alcohol was given to each participant laboratory. The adults were double-mounted on micro-pins while fresh, then preserved in insect boxes for a period not exceeding 2 years. A senior entomologist double-checked the previously identified samples before they were taken to the participating laboratories. An identification code was attributed to each specimen. Overall, 138 adult mosquitoes and 76 larvae (total = 214) were distributed to the laboratories. The precise composition of the different biological sets is given in Additional file 2: Table S2.

Although particular care was taken in handling the biological material, some specimens, especially adults, may have been damaged during transportation, a possibility not uncommon in the identification of vector specimens. Where this occurred, participants were invited to perform the identification as best they could.

Specimen sets were distributed by hand during regional meetings, which provided an opportunity to meet representatives of the target laboratories and reduced the risk of damage during transportation. The following instructions were given: (i) morphological identification should be performed on behalf of the laboratory, meaning that several persons could take part in the process; (ii) all available resources could be used to support identification (dichotomous keys, interactive keys for mosquito species, monographies and publications, molecular methods, etc.) and the choice was left to the participants’ discretion; (iii) results of the identification had to be returned within a maximum of two months *via* an online form specifying the specimen’s genus and species name.

Identification responses were classified into three categories: Correct, False and Not Responding. Responses limited to identification of the sex of adult specimens were considered False. Each head of laboratory was confidentially informed of the results of the identifications delivered by his/her laboratory. The data were anonymised prior to analysis because the aim of this study was only to evaluate the accuracy of the identifications; we did not intend to applaud, stigmatise or rank the laboratories.

## Results

**Fig. 2:**
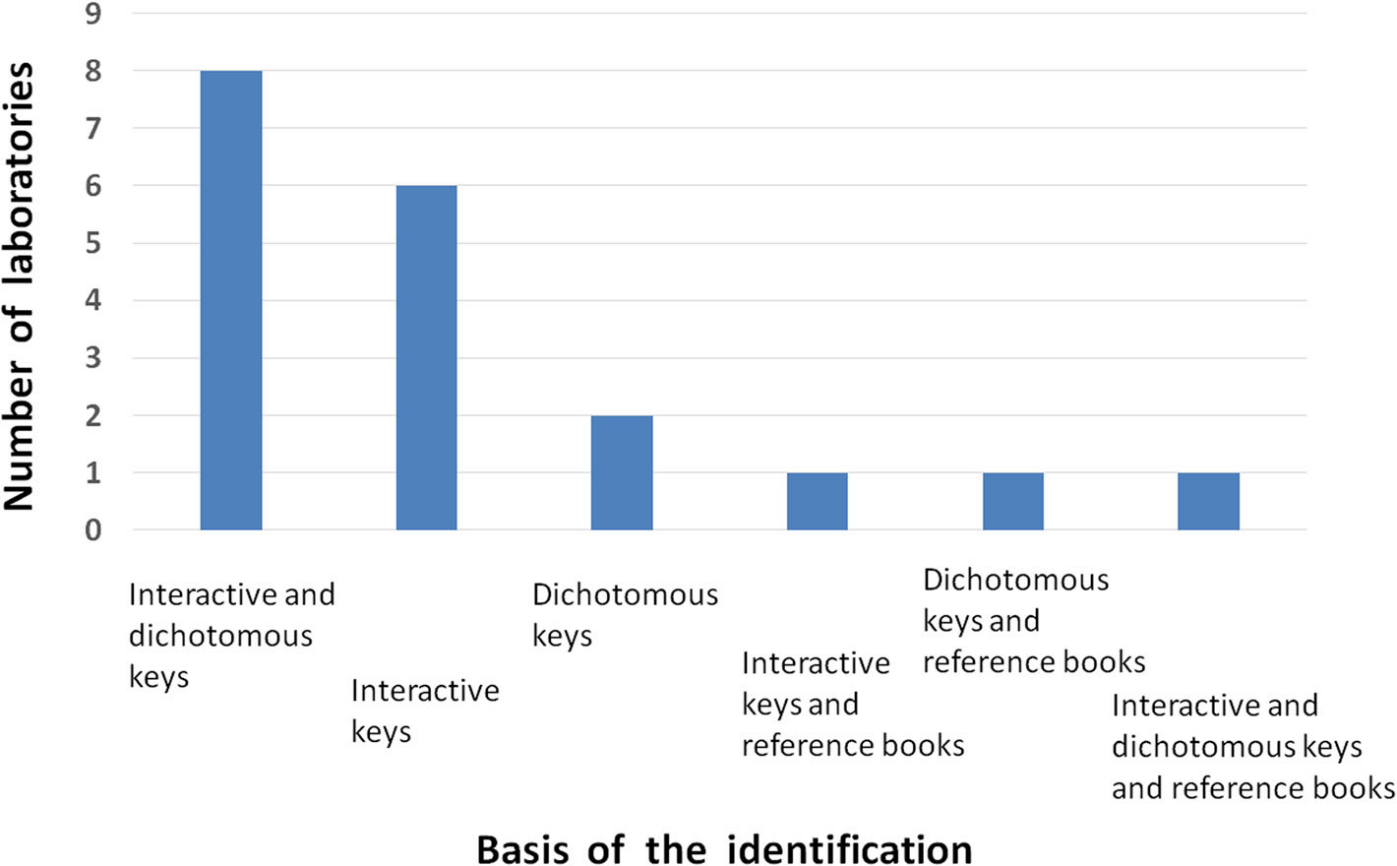
The various methodologies used to identify mosquito species according to the number of laboratories using them (19 laboratories in total)

All laboratories performed morphology-based identification using interactive identification keys / dichotomous keys / reference literature, either alone or in combination. None of them used molecular methods (PCR diagnostics, nucleic acid sequencing, MALDI-TOF, etc.). Analysis by identification tool showed that interactive keys were the most commonly used (16/19 laboratories, i.e. 84%), followed by dichotomous keys (12 laboratories; 63%), and lastly reference literature (3 laboratories; 16%). Analysis by laboratory shows that 8 laboratories (42%) relied on both interactive and dichotomous keys for identification, 6 (32%) used only interactive keys, 2 (11%) used only dichotomous keys, 1 (5%) used interactive keys and reference literature, 1 (5%) used dichotomous keys and reference literature, and 1 (5%) based its identifications on the three tools together (Fig. 2). One laboratory mentioned also using their own expertise (especially for mosquito species regularly recorded in their region) and another used web-based images to complete identification.

Eight adult specimens out of 138 (6%) were considered by the identifiers as being too damaged to allow identification, even at the genus level. This was probably due to transportation in a suitcase in the hold of an aircraft. Identification was not performed on these specimens, which were classified as Not Responding and were not included in the subsequent analysis.

Table 1 presents the percentages of correct identifications from all the respondents. Overall, 81% of identifications were correct at the genus level, while the percentage was lower (64%) at the species level. Accuracy of identification was similar for the larval and adult stages and for males and females.

**Table 1:** Identifications of mosquito genera and species, according to development stage and sex

	Percentage of correct genus identification	Fisher’s exact test (*P*-value)	Percentage of correct species identification	Fisher’s exact test (*P*-value)
All mosquitoes (*n* = 206)	81.1	–	63.6	–
All larvae (*n* = 76)	88.2	ns (0.064)	60.5	ns (0.5489)
All adults (*n* = 130)	76.9	–	65.4	–
Adult females (*n* = 96)	81.3	ns (0.060)	68.8	ns (0.2098)
Adult males (*n* = 34)	64.7	–	55.9	–

*Abbreviations*: ns, not significant

**Fig. 3:**
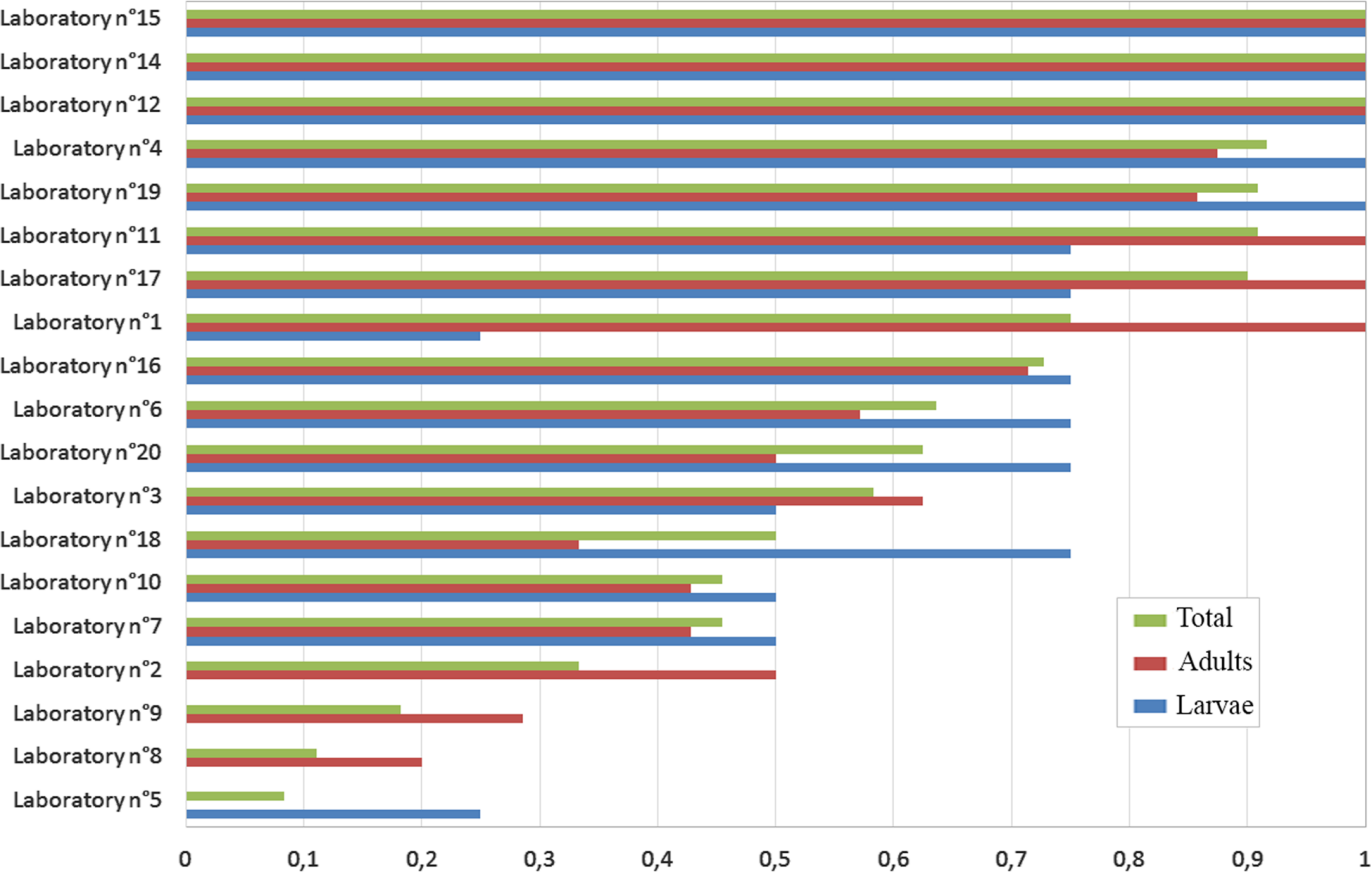
Percentages of correct identifications at the species level by country for all samples (adults and larvae), and for adults and larvae separately

The results from the different laboratories varied considerably, with accuracy of identification ranging from 100% to 8%. Three laboratories correctly identified 100% of specimens (adults and larvae) at the species level, four laboratories between 90–95%, six between and 50–75%, and six less than 50%. Details are given in Fig. 3.

Table 2 presents the results by mosquito species. The results for larvae ranged from 84% to 37% correct identifications. More than 50% of *Aedes* spp. were incorrectly identified.

**Table 2:** Identifications of mosquito species ordered by decreasing rightness of identification of adult specimens. Only species where six or more adults were correctly identified were retained for analysis

	Percentage of correct species identification (adults)	Percentage of correct species identification (larvae)
*Aedes albopictus*	95 (*n* = 19)	– (*n* = 0)
*Culiseta longiareolata*	71 (*n* = 17)	74 (*n* = 19)
*Aedes vittatus*	68 (*n* = 19)	37 (*n* = 19)
*Orthopodomyia pulcripalpis*	63 (*n* = 19)	84 (*n* = 19)
*Aedes vexans*	60 (*n* = 15)	– (*n* = 0)
*Culex hortensis*	55 (*n* = 11)	– (*n* = 0)
*Anopheles maculipennis* (*s.l.*)	50 (*n* = 6)	– (*n* = 0)
*Culex pipiens* (*s.l.*)	50 (*n* = 18)	– (*n* = 0)
*Aedes detritus* (*s.l.*)	– (*n* = 0)	47 (*n* = 19)

Regarding adults, *Aedes albopictus*, a species of some importance, was correctly identified at 95%, but another important species, *Culex pipiens* (*s.l.*), was correctly identified at 50%. As it is useful for all new mosquito taxonomists to be aware of common pitfalls, the following recurrent errors deserve to be highlighted: three Culicinae males were misidentified as Anophelinae (both have long maxillary palps); *Orthopodomyia pulcripalpis* adults were misidentified five times as *Ae. aegypti* (both have more or less similar scutum patterns); larvae of *Ae. vittatus* were identified three times as *Ae. aegypti* (both have siphon without acus or with indistinct acus); larvae of *Ae. detritus* were confused with *Ae. vexans* three times.

## Discussion

This EQA aimed to evaluate objectively the quality of mosquito identification within the MediLabSecure Network and offers useful perspectives in terms of capacity building to strengthening skills, competencies and abilities in the field of morphological identification of mosquitoes.

Taxonomy and systematics are endangered disciplines but are nonetheless a core capacity of medical entomology [15]. Reliable identification is crucial in order to support capacities for responding to vector-borne disease threats, as effective management measures can only be implemented when species are correctly identified. Misidentifications can have significant costs and consequences when decisions are needed for public health purposes. Morphological descriptions and identification keys (dichotomous or interactive) are still needed, as biological molecular tools and other “emerging” means of identification (such as MALDI-TOF mass spectrometry profiling, geometric morphometrics using landmarks on the wings, and optically-refracted wing interference patterns) are not viable alternatives [16]. However, there have been calls for decades to fill the gap in expertise in insect systematics [17], including vectors [18].

It is worth noting that, although there were no restrictions on using molecular methods, all the laboratories in our study used morphological identification alone. This may be related to the dedicated training, known as ‘MosPictoQuizz’, routinely carried out every two months within the MediLabSecure Network, in which a series of pictures of an unknown mosquito are presented for identification [19]. It may also have to do with the fact that only half the participant laboratories are properly equipped for molecular identification.

The results of this EQA highlight the great need for improving the quality and reliability of mosquito identification. Finding solutions that may help to improve the situation is a huge task because many factors interfere with the accuracy of identification, including the suitability of the key used for the mosquito (with regard to the geographical area, the development stage or the sex of the specimen), the light source, and the microscope and its adjustment to the observer and focussing, etc. The keys themselves and how they are written may also be a factor in incorrect identifications. Simple questions may check the reasonableness of identification: is it a first record in the area? If yes, is there a senior entomologist around that may confirm the identification? Is the species known to be abundant in the area and in the season of sampling? Clearly, there is a need for standards of accuracy of identification in all entomology laboratories (not just those in this network) that report mosquito vector species information.

The results of this EQA also highlight the need to pursue efforts and obtain funding for capacity building in medical entomology to improve preparedness and response to vector-borne diseases. Here, we may want to ask what the best strategy is to improve the overall situation around the Mediterranean. In our opinion, there are two key options. The first consists in increasing the training sessions in those laboratories identified as reference centres in the various countries. However, training is only a step in the process of skill acquisition, which is gradually built through personal experience and regular practice. This means that in designating reference laboratories there must be a political, or at least institutional, solution to include taxonomy as a fundamental competence wherever arthropods of medical and public health importance are dealt with. The second option would be to identify a regional reference laboratory with the capacity to deal with numerous samples and to provide expertise to the different countries in the zone. However, expertise in taxonomy requires significant investment and there is currently a global lack of such expertise. Furthermore, control would be conceded to one country in the zone, on which the others would be dependent for this type of activity with consequent loss of sovereignty.

As with any technical activity delivering results, quality control is useful to check the performance of laboratories. This type of external assessment should be repeated and extended to other methods such as molecular assays, and to other activities and processes, such as sample collection or receipt, in order to maintain standards.

## Conclusions

Identification of vectors is a core capacity in the field of medical and veterinary entomology and is needed to support response capacities to vector-borne disease threats. The results of this EQA highlight the need to maintain efforts in capacity building and quality control in the field of medical entomology and, more specifically, in the morphological identification of Culicidae.

## Additional files

Additional file 1: List of the 19 member laboratories of the MediLabSecure medical and veterinary entomology network, who took part in the EQA. (XLSX 14 kb)

Additional file 2: Detailed results of identification of the Culicidae mosquitoes, by laboratory. (XLSX 23 kb)

## Abbreviations

EQA: External quality assessment
